# Inhibition of TNF-*α* Reverses the Pathological Resorption Pit Profile of Osteoclasts from Patients with Acute Charcot Osteoarthropathy

**DOI:** 10.1155/2015/917945

**Published:** 2015-06-02

**Authors:** Nina L. Petrova, Peter K. Petrov, Michael E. Edmonds, Catherine M. Shanahan

**Affiliations:** ^1^Diabetic Foot Clinic, King's College Hospital, London SE5 9RS, UK; ^2^Department of Materials, Imperial College London, London SW7 2AZ, UK; ^3^Cardiovascular Division, King's College London, London SE5 9NU, UK

## Abstract

We hypothesised that tumour necrosis factor-*α* (TNF-*α*) may enhance receptor activator of nuclear factor-*κβ* ligand- (RANKL-) mediated osteoclastogenesis in acute Charcot osteoarthropathy. Peripheral blood monocytes were isolated from 10 acute Charcot patients, 8 diabetic patients, and 9 healthy control subjects and cultured *in vitro* on plastic and bone discs. Osteoclast formation and resorption were assessed after treatment with (1) macrophage-colony stimulating factor (M-CSF) and RANKL and (2) M-CSF, RANKL, and neutralising antibody to TNF-*α* (anti-TNF-*α*). Resorption was measured on the surface of bone discs by image analysis and under the surface using surface profilometry. Although osteoclast formation was similar in M-CSF + RANKL-treated cultures between the groups (*p* > 0.05), there was a significant increase in the area of resorption on the surface (*p* < 0.01) and under the surface (*p* < 0.01) in Charcot patients compared with diabetic patients and control subjects. The addition of anti-TNF-*α* resulted in a significant reduction in the area of resorption on the surface (*p* < 0.05) and under the surface (*p* < 0.05) only in Charcot patients as well as a normalisation of the aberrant erosion profile. We conclude that TNF-*α* modulates RANKL-mediated osteoclastic resorption *in vitro* in patients with acute Charcot osteoarthropathy.

## 1. Introduction

Charcot osteoarthropathy is a severe complication of diabetes, which is associated with significant morbidity and mortality [1–5]. Inflammation and increased osteoclastic activity are well-recognised drivers of the rapid bone destruction that occurs in the Charcot foot, although the link between them is not fully understood [6].

We have recently demonstrated that, in acute Charcot osteoarthropathy, there is increased osteoclastic activity in response to the osteoclastogenic cytokine receptor activator of nuclear factor-*κβ* ligand (RANKL) [7]. Osteoclasts, generated from peripheral blood monocytes of Charcot patients in the presence of the stimulating factor macrophage-colony stimulating factor (M-CSF) and RANKL, excessively resorb bone slices. Using the novel technique of surface profilometry, in addition to traditional light microscopy, we have shown that osteoclasts derived from Charcot patients eroded bone surfaces with an aberrant pit profile and geometry [8]. Resorption pits from cultures of Charcot patients appeared more frequently as multidented pits and were significantly deeper and wider compared with resorption pits in healthy controls [8].

The reason for this increased resorbing activity is unknown, but it is possible that it is driven by uncontrolled inflammation due to upregulation of proinflammatory cytokines and in particular tumour necrosis factor-*α* (TNF-*α*) [6]. As an osteoclastogenic mediator, TNF-*α* induces expression of RANKL in osteoblastic cells, but it can also act directly on osteoclastic precursors (monocytes) to potentiate RANKL-induced osteoclastogenesis and thereby activity [9]. This cytokine is known to enhance osteoclastogenesis in rheumatoid arthritis [10, 11] and psoriatic arthritis [12] and also in other forms of inflammatory osteolysis [13] and we hypothesised that TNF-*α* may also modulate osteoclastic activity in acute Charcot osteoarthropathy. Thus the aim of this study was to determine the role of this cytokine by comparing the extent of osteoclast formation and resorption in M-CSF + RANKL-treated cultures with and without the addition of neutralising antibody to TNF-*α* (anti-TNF-*α*).

## 2. **Materials and Methods**

### 2.1. Patients

Samples from peripheral blood were obtained from 10 consecutive patients with recent onset of acute Charcot osteoarthropathy, 8 diabetic patients with no history of Charcot osteoarthropathy, and 9 healthy control subjects. All patients with Charcot osteoarthropathy presented with a unilateral red hot swollen foot and radiological evidence of acute Charcot fractures, demonstrated on plain foot and ankle radiographs [1, 5]. All participants had intact feet and had no features of foot infection or sepsis. The study was approved by the Outer West London Research Ethics Committee and was carried out in accordance with institutional guidelines and the Declaration of Helsinki with all patients and control subjects signing written informed consent.

### 2.2. Isolation and Culture of Peripheral Blood Mononuclear Cells (PBMCs)

Peripheral blood mononuclear cells (PBMCs) were isolated from whole blood as previously described [8]. The PBMCs were separated after gradient centrifugation and resuspended in culture medium and 2 × 10^6^ cells were cultured on 24-well plates and 5 × 10^5^ cells were cultured on bovine bone discs (Immunodiagnostic Systems Ltd., Boldon, UK) in duplicate to assess osteoclast formation and resorption, respectively. Cultures were maintained in *α*-minimal essential medium (*α*-MEM, Lonza, Wokingham, UK) supplemented with penicillin (50 U/mL)/streptomycin (50 *μ*g/mL) (Sigma-Aldrich Ltd., Poole, UK), L-glutamine (2 mM) (Sigma-Aldrich Ltd., Poole, UK), and 10% heat-inactivated FBS (Lonza Ltd., Wokingham, UK) under the following conditions:

- Cultures with M-CSF 25 ng/mL (added at day 0) (R&D Systems Europe, Ltd., Abingdon, UK) and soluble RANKL 100 ng/mL (added at day 7) (PeproTech EC Ltd., London, UK): M-CSF + RANKL-treated cultures served as a positive control.
- Cultures with M-CSF (added at day 0), anti-TNF-*α* 10 *μ*g/mL (added at day 0), (R&D Systems Europe, Ltd., Abingdon, UK), and soluble RANKL 100 ng/mL (added at day 7): M-CSF + RANKL + anti-TNF-*α*-treated cultures were used to assess the role of TNF-*α* on osteoclastogenesis. The rationale for this study was to inhibit TNF-*α* modulation on peripheral blood monocytes by using excess concentration of anti-TNF-*α*, added from the beginning until the end of the cell culture treatment [14].

Culture medium was refreshed every 3-4 days supplemented with the appropriate agents as described above. After 17 days in culture, 24-well plates were stained for tartrate-resistant acid phosphatase (TRAP). Plates were viewed by light microscopy and TRAP-positive cells with three or more nuclei were counted as osteoclasts. The ability of these cells to resorb bone was demonstrated by culturing PBMCs on bovine bone discs for 21 days. The bone discs were stained with toluidine blue and mounted on glass slides. Resorption was quantitated by two methods: (1) area of resorption on the surface (%) assessed by image analysis after light microscopy and (2) area of resorption under the surface (*μ*m^2^) assessed by surface profilometry, as previously described [8]. The erosion profile of resorbed bone discs was measured by the Dektak 150 Surface Profiler (Veeco, New York, USA) fitted with a stylus (radius 2.5 *μ*m), as previously described [8]. The stylus was dragged across the surface of the sample in hills and valleys mode with ten scans per subject carried out at random sites on each of the two discs. Each measurement had the following scan parameters: stylus force: 3.00 mg, scan length: 1000 *μ*m, scan duration: 60 seconds, vertical measurement range: 65.5 *μ*m, scan resolution: 0.056 *μ*m/scan. On average, 75 pits per condition/per subject were analysed and the median area of disc erosion was calculated in *μ*m^2^ using Origin Pro 8.6 software.

According to their shape, pits were defined as unidented (erosion with one dent starting from and finishing at the level of the unresorbed surface), bidented (erosion with two clearly defined dents starting from and finishing at the level of the unresorbed surface), and multidented (erosion with three or more dents starting from and finishing at the level of the unresorbed surface). Each pit was characterised by the following parameters: width at the surface (*μ*m), maximum depth (*μ*m), and full-width-half-maximum (FWHM) (*μ*m), where the width was measured at the half of the maximum depth. The median width, depth, and FWHM for the unidented, bidented, and multidented pits were calculated for each subject [8].

### 2.3. Statistical Analyses

Data were analysed with Predictive Analytics Software 18 statistical package and expressed as median (25th–75th percentile). Differences between study groups and culture treatments were analysed using the nonparametric Mann-Whitney *U* test (two groups) or Kruskal-Wallis test (three groups), as appropriate. Chi-square test was used for categorical variables. Differences were considered significant at *p* < 0.05.

## 3. Results

### 3.1. Demographical Features

Patients with acute Charcot osteoarthropathy were matched for age, gender, and type and duration of diabetes with the diabetic patients and for age and gender with the healthy control subjects. The age, gender distribution, and type and duration of diabetes were not significantly different between the Charcot patients and diabetic patients nor were the age and gender distribution between the Charcot patients and healthy control subjects (Table 1).

### 3.2. Osteoclast Formation

**Figure 1:**
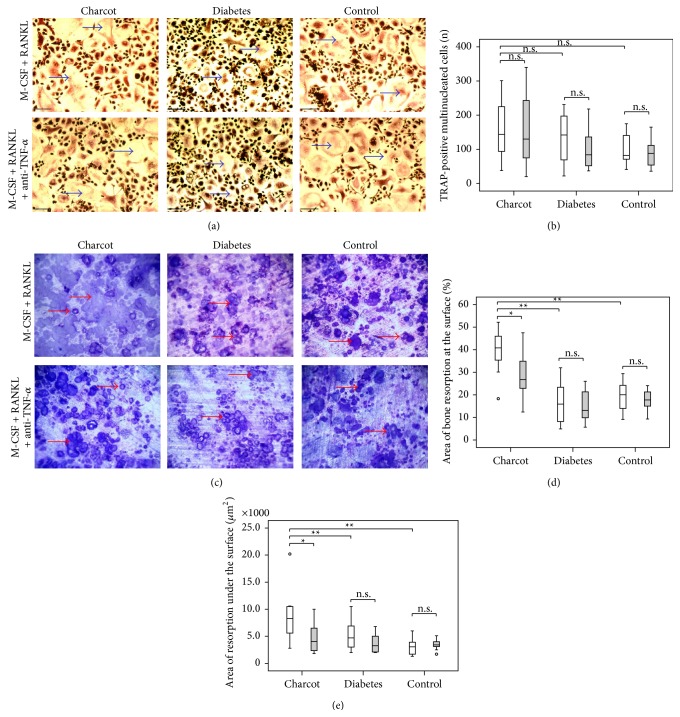
Osteoclast formation and resorption in Charcot patients, diabetic patients, and healthy control subjects in M-CSF + RANKL-treated cultures and in M-CSF + RANKL + anti-TNF-*α*-treated cultures. Representative images of TRAP-positive multinucleated cells formed on plastic (Olympus; original magnification ×100, scale bar = 200 *μ*m) (a) and resorbed bovine bone discs (Olympus BX60; original magnification ×200) (c). The arrows denote some of the TRAP-positive multinucleated cells (a) and some of the resorption pits (c). Comparison of the number of TRAP-positive multinucleated cells (b), the area of resorption at the surface (d), and the area of resorption under the surface (e) of resorbed bone discs between M-CSF + RANKL-treated cultures (white bars) and M-CSF + RANKL + anti-TNF-*α*-treated cultures (grey bars). Significance assessed by Mann-Whitney *U* test, levels of significance are demonstrated on the graphs; ^*∗*^ *p* < 0.05; ^*∗∗*^ *p* < 0.01; ns = nonsignificant (*p* > 0.05).

Observation of the cell culture plates with light microscopy showed no difference in osteoclast formation in M-CSF + RANKL-treated cultures between the three groups (Figure 1(a)). The median number of TRAP-positive multinucleated cells in M-CSF + RANKL-treated cultures in Charcot patients was not significantly different from the median number of TRAP-positive multinucleated cells in diabetic patients and healthy control subjects (Figure 1(b)).

The addition of anti-TNF-*α* to M-CSF + RANKL treatment did not lead to a significant difference in the median number of TRAP-positive multinucleated cells in Charcot patients, diabetic patients, and healthy control subjects (Figures 1(a) and 1(b)).

### 3.3. Osteoclast Resorption

Traditional light microscopy (Figure 1(c)) together with surface profilometry revealed that the newly formed osteoclasts isolated from patients with acute Charcot osteoarthropathy exhibited increased resorbing activity in M-CSF + RANKL-treated cultures compared with osteoclasts generated from diabetic patients and healthy controls, as indicated by a significantly increased area of resorption on the surface (Figure 1(d)) and under the surface (Figure 1(e)).

The addition of anti-TNF-*α* to M-CSF + RANKL treatment led to a significant reduction in the area of resorption on the surface (Figures 1(c) and 1(d)) and under the surface (Figure 1(e)) only in Charcot patients but not in diabetic patients or healthy control subjects. In Charcot patients, the area of resorption on the surface assessed by image analysis was 30% smaller in M-CSF + RANKL + anti-TNF-*α*-treated cultures compared with M-CSF + RANKL-treated cultures (Figure 1(d)) as was the area of resorption under the surface after surface profilometry (Figure 1(e)).

### 3.4. Erosion Profile of Resorbed Bovine Bone Discs after Surface Profilometry

**Figure 2:**
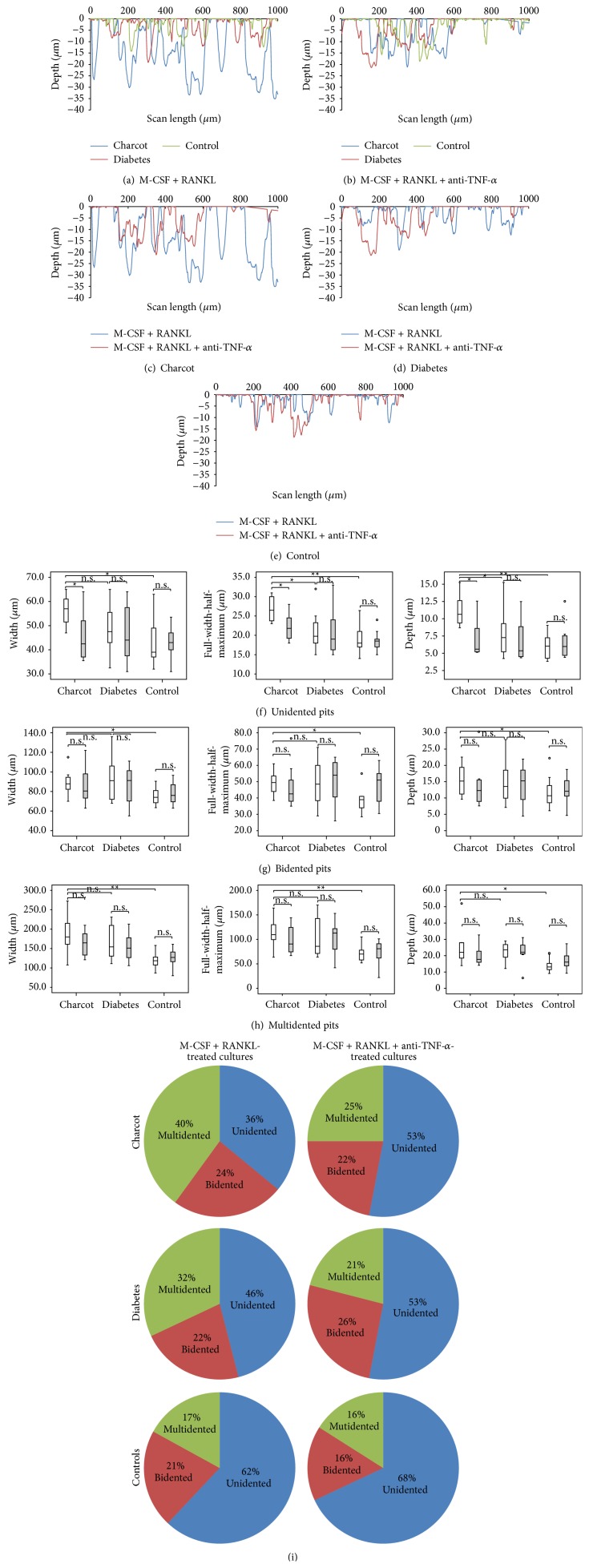
Surface profilometry in Charcot patients, diabetic patients, and healthy control subjects in M-CSF + RANKL-treated cultures and in M-CSF + RANKL + anti-TNF-*α*-treated cultures. Representative erosion profiles of resorbed bone discs in Charcot patient (blue line), diabetic patient (red line), and healthy control subject (green line) after surface profilometry in M-CSF + RANKL-treated cultures (a) and in M-CSF + RANKL + anti-TNF-*α*-treated cultures (b). Representative erosion profiles of resorbed bone discs in M-CSF + RANKL-treated cultures (blue line) and M-CSF + RANKL + anti-TNF-*α*-treated cultures (red line) in Charcot patient (c), diabetic patient (d), and healthy control subject (e). The marked difference of the erosion profile in a Charcot patient compared with diabetic patient and healthy control in MCSF + RANKL-treated cultures (a) was reversed after the addition of anti-TNF-*α* (b). Pits appeared significantly smaller in M-CSF + RANKL + anti-TNF-*α*-treated cultures compared with MCSF + RANKL-treated cultures in Charcot patients (c) but remained unchanged in diabetic patients (d) and in healthy controls (e). Comparison of pit measurements (width, FWHM, and depth) between M-CSF + RANKL-treated cultures (white bars) and M-CSF + RANKL + anti-TNF-*α*-treated cultures (grey bars); unidented pits (f), bidented pits (g), and multidented pits (h). Significance assessed by Mann-Whitney *U* test, levels of significance are demonstrated on the graphs; ^*∗*^ *p* < 0.05; ^*∗∗*^ *p* < 0.01; ns = nonsignificant (*p* > 0.05). Distribution of pits (%) according to their shape in M-CSF + RANKL and M-CSF + RANKL + anti-TNF-*α*-treated cultures in Charcot patients, diabetic patients, and healthy control subjects (i). In M-CSF + RANKL-treated cultures, there was a significant reduction in the percentage of unidented (*p* < 0.05) and a significant increase in the percentage of multidented pits (*p* < 0.05) in Charcot patients compared with diabetic patients and healthy controls. The addition of anti-TNF-*α* treatment led to a significant difference in the pit distribution only in Charcot patients characterised by a significant increase in the percentage of unidented pits (*p* < 0.05) and significant decrease in the percentage of multidented pits (*p* < 0.05). No differences in the pit distribution were noted in diabetic patients and in healthy control subjects.

The surface profile measurements of randomly selected areas revealed multishaped erosions of resorbed bovine bone discs in all study groups in both culture treatments (Figures 2(a) and 2(b)).

However, in M-CSF + RANKL-treated cultures, the erosion profiles were markedly different between the three groups (Figure 2(a)). The erosions appeared greater and deeper in Charcot patients compared to erosions in diabetic patients and healthy controls (Figure 2(a)).

After the addition of anti-TNF-*α* to M-CSF + RANKL treatment, the observed differences in the erosion profile between the three groups were lost (Figure 2(b)). In Charcot patients, there was a “normalisation” of the erosion profile in M-CSF + RANKL + anti-TNF-*α*-treated cultures compared to M-CSF + RANKL-treated cultures (Figure 2(c)), whereas anti-TNF-*α* had no effect on the erosion profiles of diabetic patients (Figure 2(d)) or healthy control subjects (Figure 2(e)).

### 3.5. Pit Morphology

To assess in more detail the differences in resorption under the surface, pit morphology was evaluated. In M-CSF + RANKL-treated cultures, the pit parameters (median width, FWHM and depth) were greater in Charcot patients compared with diabetic patients and healthy control subjects (Figures 2(f), 2(g), and 2(h)). The addition of anti-TNF-*α* to M-CSF + RANKL treatment led to a significant reduction in the width, FWHM, and depth of the unidented pits in Charcot patients (Figure 2(f)). Although the reduction of the bidented pit parameters (Figure 2(g)) and multidented pit parameters (Figure 2(h)) was not significant, there was a general trend of “normalisation” of pits after anti-TNF-*α*. In contrast to Charcot patients, the addition of anti-TNF-*α* had no effect on pit parameters in diabetic patients or in healthy control subjects (Figures 2(f), 2(g), and 2(h)).

### 3.6. Pit Distribution

To determine whether there were any differences in the distribution of the shape of the pits, the percentage of unidented, bidented, and multidented pits between the two culture treatments was compared. In Charcot patients, the addition of anti-TNF-*α* to M-CSF + RANKL resulted in a significant increase in the percentage of unidented pits (from 36% [31–43] to 53% [43–63], *p* < 0.05) and a significant decrease in the percentage of multidented pits (from 40% [32–42] to 25% [13–33], *p* < 0.05), whereas the percentage of bidented pits remained unchanged (from 24% [20–28] to 22% [21–24],  *p* > 0.05) (Figure 2(i)). There was no significant difference in the distribution of pits (unidented, bidented, and multidented) between the two culture treatments in the diabetic patients and in the healthy controls (Figure 2(i)).

## 4. Discussion

This* in vitro* study has shown that there was a significant reduction in the resorbing activity of M-CSF + RANKL-treated osteoclasts derived from Charcot patients in response to anti-TNF-*α* treatment. The addition of anti-TNF-*α* resulted in significant reduction in the area of resorption on bovine bone discs both on the surface, as assessed by image analysis, and also under the surface, as assessed by surface profilometry. The aberrant erosion profile, pit morphology, and pit distribution in M-CSF + RANKL-treated cultures in Charcot patients were reversed after the addition of anti-TNF-*α*. These findings confirm the hypothesis that the proinflammatory cytokine TNF-*α* modulates increased osteoclastic activity in acute Charcot osteoarthropathy.

In the present study, we have shown that newly derived osteoclasts from monocytes isolated from Charcot patients exhibit an enhanced response to RANKL. Osteoclastogenesis is pivotally dependant on M-CSF (a survival factor) and RANKL (key factor for osteoclast differentiation and regulation) [15, 16]. In the presence of these two cytokines, monocytes, which express the receptors c-fms and RANK, proliferate and differentiate into mature multinucleated osteoclasts [17, 18]. The osteoclasts generated from Charcot patients were functionally more aggressive compared to osteoclasts from diabetic patients and healthy control subjects in a classical resorption assay [7, 8]. In addition, surface profilometry demonstrated that osteoclasts from Charcot patients exhibited a considerable below-surface resorbing activity, which was not associated with an increase in osteoclast formation [8]. However, the mechanisms of this enhanced response are unknown.

In Charcot osteoarthropathy bone loss is limited to the inflamed affected foot [19, 20] and it is possible that local inflammatory factors released after initial trauma to the Charcot foot may act as osteoclastogenic mediators [6]. In this study, we sought to determine the role of TNF-*α* as a promoter of the observed enhanced osteoclast function. This cytokine has been linked with inflammatory bone loss [12] and immunohistochemical analysis of surgical Charcot specimens has indicated that osteoclastic bone resorption takes place in the presence of TNF-*α* [21]. In the acute stage of the osteoarthropathy, serum concentrations of TNF-*α* are raised [22]. Moreover, in the acute Charcot foot, inflammatory modulation of peripheral monocytes with increased spontaneous and induced production of TNF-*α* has been noted [23]. In this study, using the traditional resorption pit assay together with surface profilometry, we have demonstrated that although osteoclast formation remained unchanged, the addition of anti-TNF-*α* to M-CSF + RANKL-treated cultures significantly decreased osteoclast function. This is in agreement with previous data showing that TNF-*α* is more potent for osteoclast activation than for osteoclast formation [24]. The inhibition of TNF-*α* led to a significant reduction in the area of resorption on the surface and under the surface in cultures from Charcot patients.

As well as increased resorption, in our study, there were a pathological erosion profile and aberrant morphological appearance of resorption pits on bone slices in M-CSF + RANKL-treated cultures in Charcot patients compared with healthy control subjects [8] and also in Charcot patients compared with diabetic patients. We demonstrated that the addition of anti-TNF-*α* reversed the observed differences in pit parameters and erosion profile between the study groups and in Charcot patients there was a notable “normalisation” of the erosion profile and pit morphology. This suggests that osteoclasts generated in M-CSF + RANKL-treated cultures, prior to inhibition of TNF-*α*, exhibit a highly aggressive resorptive profile. This exuberant resorptive activity was reduced after the addition of anti-TNF-*α*, providing further evidence to support the role of this cytokine in the osteoclastogenesis of acute Charcot osteoarthropathy.

As well as changes in the morphological appearance of pits, there was a difference in the distribution of the shape of the pits in Charcot patients between the two culture treatments. The resorption pits in M-CSF + RANKL-treated cultures of the Charcot patients were predominantly multidented and bidented whilst the unidented pits were less frequently seen although in diabetic patients and in healthy control subjects the pit distribution remained unchanged between the two culture treatments.

**Figure 3:**
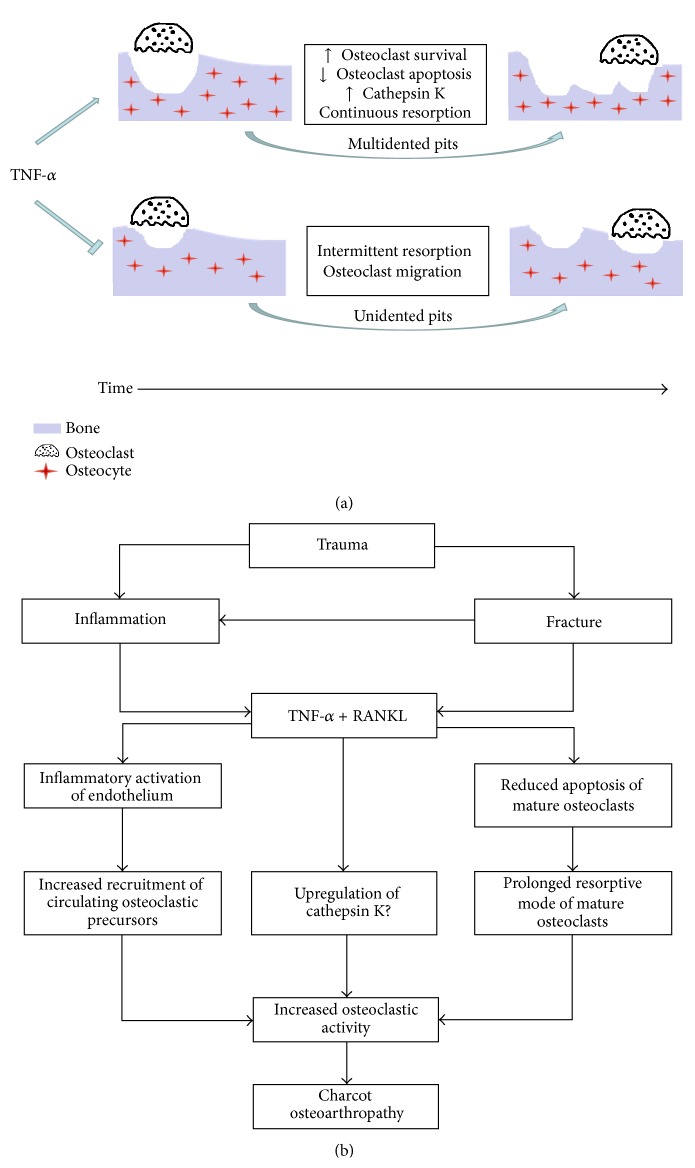
Model for osteoclast resorptive activity in Charcot osteoarthropathy in M-CSF + RANKL-treated cultures before and after the addition of anti-TNF-*α* (a). In the presence of TNF-*α*, continuous multidented pits (defined as lacunae or trenches) with increased area of resorption both on the surface and under the surface are seen more frequently compared with less frequently noted unidented pits (single resorption event). Resorption is prolonged and is not interrupted by migration episodes. After the inhibition of TNF-*α*, the percentage of multidented pits is reduced with a corresponding increase in the percentage of unidented pits suggesting that the resorptive cycle is restored. Resorption alternates with migration and intermittent resorption events occur away from each other (unidented pits). The observed differences in the resorption before and after the addition of anti-TNF-*α* suggest that TNF-*α* (via cathepsin K upregulation) modulates the resorptive behaviour of osteoclasts generated from Charcot patients and these highly active osteoclasts are capable of extensive lacunar resorption with aberrant pit morphology and geometry due to reduced migration, increased survival, and reduced apoptosis. The proposed role of TNF-*α* in the pathogenesis of pathological bone destruction in the acute Charcot foot (b).

The addition of anti-TNF-*α* to M-CSF + RANKL-treated cultures resulted in a significant reduction in the percentage of the multidented pits as well as a significant increase the percentage of the unidented pits. Thus, the inhibition of TNF-*α* normalised the resorptive behaviour of Charcot osteoclasts in which resorption alternated with migration as indicated by a significant increase in the percentage of unidented pits. It is possible that observed aberrant pit morphology and distribution were due to a TNF-*α* modulation of the resorption cycle (Figure 3(a)). During the process of bone resorption, osteoclasts solubilise bone mineral followed by degradation of demineralised organic matrix and in control conditions, the relative rate of collagenolysis is slower than the rate of demineralisation [25]. Experimental* in vitro* studies have shown that agents which can upregulate cathepsin K expression prolong the resorption cycle and resorption events more frequently present as trenches (continuous resorption) [25]. In contrast, inhibition of cathepsin K accelerates the resorption cycle, leading to faster accumulation of collagen. This results in resorption events more frequently presenting as shallower and smaller pits (intermittent resorption). Both RANKL and TNF-*α* stimulate the osteoclasts to produce cathepsin K, which is the major protease responsible for the degradation of collagen [26]. In Charcot osteoarthropathy it is possible that TNF-*α* via enhanced cathepsin K expression may lead to imbalance between the relative rate of collagenolysis and demineralisation. This mechanism may explain the continuous resorptions which we observed as multidented pits in the M-CSF + RANKL-treated cultures in contrast to the more frequently noted intermittent resorptions (unidented pits) after the addition of anti-TNF-*α* [25] (Figure 3(a)).

These findings have important implications for understanding the pathogenesis of this condition. Our data underscores the potent role of TNF-*α* in the RANKL-mediated osteoclastogenesis (Figure 3(b)). Trauma to the neuropathic diabetic foot leads to bone damage and uncontrolled inflammation [6]. Bone fracture is the harbinger of Charcot osteoarthropathy [27] and it leads to changes in the bone matrix, which becomes a site of targeted remodelling with increased numbers of apoptotic osteocytes (bone matrix cells) and rapid degradation by activated osteoclasts [18, 28]. Furthermore, bone fracture triggers a coordinated healing cytokine response with the induction of proinflammatory cytokines, including TNF-*α* [29]. In the affected Charcot foot, the inflammatory response to trauma with increased cytokine release leads to an upregulation of receptors and adhesion molecules in the endothelium which then forms a firm attachment to osteoclast precursors resulting in enhanced recruitment of osteoclasts to sites of bone resorption [30]. Furthermore, the activation of RANK by RANKL attracts osteoclastic precursors [31] and its upregulation contributes to an enhanced RANKL-induced monocyte migration to the affected foot. Thus TNF-*α*-primed osteoclastic precursors in the presence of increased local expression of RANKL differentiate into highly aggressive osteoclasts with extensive resorbing activity characterised by increased survival and reduced apoptosis and migration. This increased osteoclastic activity may be due to cathepsin K upregulation, which requires further studies, as this may provide scientific basis for novel intervention with cathepsin K inhibitors. Overall, these aberrantly activated osteoclasts play a key role in the pathological bone destruction of the acute Charcot foot.

In conclusion, using a traditional osteoclast resorption assay together with surface profilometry, this study has demonstrated for the first time that the proinflammatory cytokine TNF-*α* modulated RANKL-mediated osteoclastic resorption* in vitro* in patients with acute Charcot osteoarthropathy and these observations shed light into the pathogenesis of this devastating condition.

## Figures and Tables

**Table 1:** Demographic features of the study patients.

	Charcot	Diabetes	Healthy control subjects
Age (years)	57 [53–64]	60 [55–66]	45 [42–48]
Gender (male : female)	6 : 4	3 : 4	5 : 3
Type 1 : type 2 diabetes	4 : 6	1 : 6	—
Duration of diabetes (years)	17 [8–29]	10 [9–26]	—

Data expressed as median [25th–75th percentile].

Nonsignificant difference in age, gender distribution, type and duration of diabetes (Charcot patients versus diabetic patients), and age and gender (Charcot patients versus healthy control subjects); (*p* > 0.05 for all pairwise comparisons).
